# Disruption of *OsEXO70A1* Causes Irregular Vascular Bundles and Perturbs Mineral Nutrient Assimilation in Rice

**DOI:** 10.1038/srep18609

**Published:** 2015-12-22

**Authors:** Bin Tu, Li Hu, Weilan Chen, Tao Li, Binhua Hu, Ling Zheng, Zheng Lv, Shuju You, Yuping Wang, Bingtian Ma, Xuewei Chen, Peng Qin, Shigui Li

**Affiliations:** 1 Rice Research Institute of Sichuan Agricultural University, Chengdu Wenjiang, Sichuan, 611130, PR China; 2 State Key Laboratory of Hybrid Rice, Sichuan Agricultural University, Chengdu Wenjiang, Sichuan, 611130, PR China; 3 Hybrid rice research center of Neijiang academy of agricultural, Neijiang, Sichuan, 641000, PR China

## Abstract

Normal uptake, transportation, and assimilation of primary nutrients are essential to plant growth. Tracheary elements (TEs) are tissues responsible for the transport of water and minerals and characterized by patterned secondary cell wall (SCW) thickening. Exocysts are involved in the regulation of SCW deposition by mediating the targeted transport of materials and enzymes to specific membrane areas. EXO70s are highly duplicated in plants and provide exocysts with functional specificity. In this study, we report the isolation of a rice mutant *rapid leaf senescence2* (*rls2*) that exhibits dwarfism, ferruginous spotted necrotic leaves, decreased hydraulic transport, and disordered primary nutrient assimilation. Histological analysis of *rls2-1* mutants has indicated impaired cell expansion, collapsed vascular tissues, and irregular SCW deposition. Map-based cloning has revealed that *RLS2* encodes OsEXO70A1, which is one of the 47 members of EXO70s in rice. *RLS2* was widely expressed and spatially restricted in vascular bundles. Subcellular localization analysis demonstrated that RLS2 was present on both membrane and nuclear regions. Expression analysis revealed that mutations in *rls2* triggers transcriptional fluctuation of orthologous EXO70 genes and affects genes involved in primary nutrient absorption and transport. In brief, our study revealed that *RLS2* is required for normal vascular bundle differentiation and primary nutrient assimilation.

Primary nutrients, including nitrogen (N), phosphorus (Pi), and potassium ion (K^+^), are essential for plant growth and are required in larger quantities than other nutrients. The mechanism of absorption, transportation, and assimilation of these three primary nutrients has been extensively studied in *Arabidopsis* and rice^1^,^2^,^3^,^4^,^5^,^6^,^7^. *PHO2*, miR399, and *PHR1* are involved in plant Pi signaling. *PHR1* is activated by Pi deficiency and promotes the accumulation of miR399, which subsequently regulates the UBC protein *PHO2* at the transcription level. PHO2 and NLA-mediated ubiquitination monitors the expression of a subset of phosphate starvation-induced (PSI) genes, including Pi transporter genes^8^,^9^,^10^. Plants use mainly inorganic nitrogen nitrate in aerobic uplands and use ammonium in flooded anaerobic paddy fields. Members of the *NRT2* family play a major role in nitrate uptake^11^,^12^, whereas those of the *OsAMT1* family are involved in NH_4_^+^ transport in rice plants^13^,^14^. K^+^ is the most abundant cation in living cells and maintains cellular electroneutrality and osmotic equilibrium. Transport of K^+^ from the soil to its final destination in plants is mediated by channels and transporters. K^+^ channels are not restricted to the plasma membrane (PM), but are also widely distributed across other membrane systems*. Os-AKT1*, which is thought to be the most important component that is involved in K^+^ uptake in rice roots, is located in the PM^6^. Pore K^+^ (*TPK*) channel family genes, which encode vacuole-specific K^+^ transporters, play an important role in maintaining K^+^ homeostasis^5^. K^+^ efflux antiporter (*KEA*) genes encode putative potassium efflux antiporters that are mainly located in the chloroplast^15^. However, how integral membrane proteins arrive at specific destinations in plants is unknown.

Tracheary elements (TEs), which are responsible for the transport of water and minerals in terrestrial plants, are characterized by secondary cell wall (SCW) thickening that present an elaborate pattern^16^. Deposition of lignified secondary walls in TEs facilitates waterproofing for efficient water transport and promotes resistance to the negative pressure generated during transpiration. It is well known that cortical microtubules play crucial roles in the patterned deposition of secondary walls^17^. The observation of fluorescent protein-fused CesAs reveals that CesA complexes move along the track of cortical microtubules located beneath the PM^18^. *AtKTN1* encodes a katanin microtubule-severing protein that is essential for the organization of cortical microtubules, and a mutation in this gene causes aberrant orientation of cellulose microfibrils^19^,^20^. Cortical microtubule bundles control the patterned deposition of secondary walls by directing the targeted transport of vesicles carrying materials and enzymes to specific PM domains^21^,^22^.

The tethering complexes, which are assembled with eight proteins (Sec3, Sec5, Sec6, Sec8, Sec10, Sec15, Exo70, and Exo84) on the vesicle membrane mediate the docking of secretory vesicles on the target membrane^23^,^24^. Bioinformatics analysis has identified homologs corresponding to these eight exocyst proteins in plants^25^,^26^,^27^. Interestingly, compared to the single copy that predominates in other eukaryotes, a striking feature of plants is that exocyst complex genes have evolved multiple paralogs^25^,^28^. Plant genomes encode a large number of EXO70 copies, for example, 23 EXO70 copies exist in *Arabidopsis thaliana*, whereas 47 occur in rice (*Oryza sativa*)^28^. Investigations of plant *EXO70* family genes are in its early stages and only a few *EXO70* paralogs have been reported in *Arabidopsis*. The distinct and tissue-specific expression patterns of *EXO70* members are suggestive of its functional divergence and specificity in regulating cell-type specific exocytosis or cargo-specific exocytosis^26^,^28^,^29^. The expression of *EXO70B2* and *EXO70H1* are upregulated during *Pseudomonas syringae* infection and are involved in the plant-pathogen interaction in *Arabidopsis*^30^,^31^. *EXO70C1* is highly expressed in guard cells and pollen grains, and is critical for pollen tube growth^32^. A recent report indicated that *EXO70H4* is essential for trichome development in both *Arabidopsis* and cucumber^33^. *EXO70E2* has been implicated in distinctive exocytotic organelles, namely EXPO, which mediate cytosol to cell wall exocytosis^34^. Among the *EXO70* paralogs, *EXO70A1* has been well characterized. *EXO70A1* is expressed in most tissues, except for mature pollen^28^,^35^,^36^,^37^,^38^,^39^. *EXO70A1* has been implicated in a wide range of developmental processes, including elongation of hypocotyls, formation of stigmatic papillae, polarized secretion in elongating root hairs^28^,^40^, pollen-stigma interaction during self-incompatibility response^41^, cell plate formation^42^, pectin deposition during seed coats development^36^, and auxin polar transport in root epidermal and cortical cells^43^. Recent studies have shown that *EXO70A1* is primarily expressed in TEs and regulate vesicle trafficking during TE differentiation to mediate patterned secondary cell wall thickening in *Arabidopsis thaliana*^37^. Therefore, EXO70A1 is an essential subunit for exocytosis and is required for proper cell wall development in *Arabidopsis*. However, no *EXO70* homologs in rice have been reported and thus its functions remain unclear. In this study, we identified the rice *RLS2* gene that encodes OsEXO70A1, a key subunit of exocysts. Mutation in the *rls2* causes irregular vascular bundles, abnormal SCW thickening in TEs, and perturbs the assimilation of primary nutrients. Primary nutrient transporter or channel gene expression were tissue-specifically regulated in *rls2* mutants, thus suggesting the possibility that *RLS2*-mediated vesicle trafficking is responsible for the translocation of integral membrane proteins to specific destinations. In sum, the present study has revealed that RLS2 is essential for vascular bundle differentiation and mineral nutrient assimilation, and provides information on the functional complexity of exocysts in rice.

## Results

### The *rls2* mutant shows pleiotropic defects

**Figure 1:**
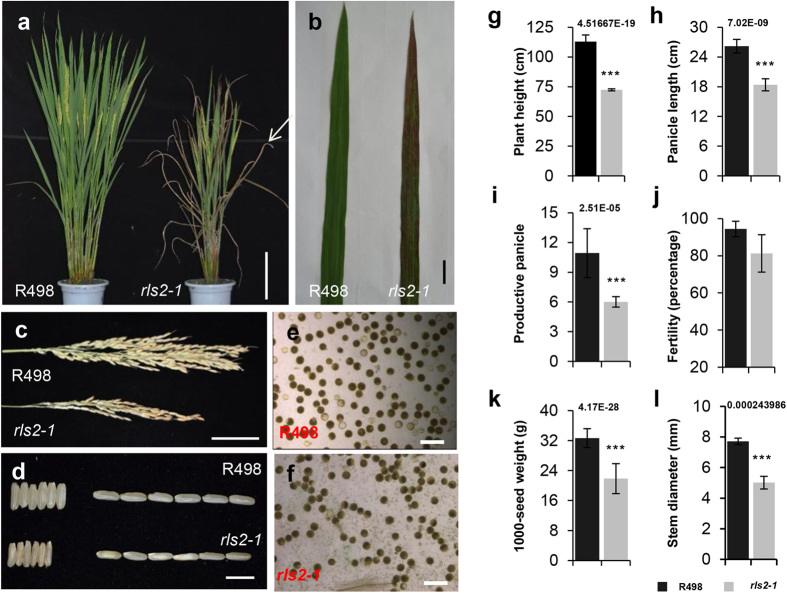
Phenotypes of field-grown wild-type (WT) and *rls2-1* mutant plants. (**a**–**f**) Performance of whole plants (**a**), leaf with ferruginous spotted necrosis (**b**), panicle length (**c**), width and length of brown rice (**d**), I-IK staining pollens in WT and *rls2-1* mutant plants (**e**,**f**). (**a**,**b**) grown in the field for 90 days. Bars = 25 cm in (**a**), 1 cm in (**b**), 5 cm in (**c**), 10 mm in (**d**), 40 μm in (**e**,**f**). (**g**–**l**) Agronomic traits (plant height, panicle length, productive panicle, fertility, 1,000-seed weight, and stem diameter) of WT (black bars) and *rls2-1* mutant (gray bars) plants. Error bars indicate SD (n = 15). p values determined by student’s *t*-test using R498 as control, ***p ≤ 0.001.

We obtained the mutant *rapid leaf senescence 2* (*rls2*) by phenotypic screening of an ethyl methane sulfonate (EMS)-induced rice mutant collection in an *indica* cultivar. The *rls2* mutant showed pleiotropic phenotypes, and its most striking abnormality was the ferruginous necrotic spots on fresh leaves, which subsequently intensified and finally led to a dessicated appearance in mature leaves (Fig. 1a,b). In addition to necrotic leaves, *rls2* plants exhibited dwarfism throughout its growth and development. Fifteen-day-old seedlings of *rls2* mutants showed reduced height, but no distinct abnormalities were observed in its roots (Fig. S1). At the mature stage, a maximum disparity in plant height was observed between the *rls2* mutant and wild-type plants (Fig. 1a and S1). The reduced height resulted from uniformly shortened internodes in the mutant culms, which were confirmed by comparison of the length of each internode between the mutant and wild-type (Fig. S1). Significant alterations were also observed in other agronomic traits such as panicle length, 1,000-grain weight, and stem diameters (Fig. 1c–l). In sum, the *rls2* mutants present necrotic leaves and overall growth abnormalities. We later acquired an allelic mutant line from the National Center of Plant Gene Research (Wuhan), in which *RLS2* expression was downregulated by T-DNA insertion into the 3’-untranslated region (3’-UTR) (see below). Therefore, these two mutant lines were named *rls2-1* and *rls2-2*, respectively. We primarily focused on the *rls2-1* mutant line in the subsequent experiments.

### The *rls2-1* mutant showing reduced hydraulic conductance and disruptions in primary nutrient assimilation

**Figure 2:**
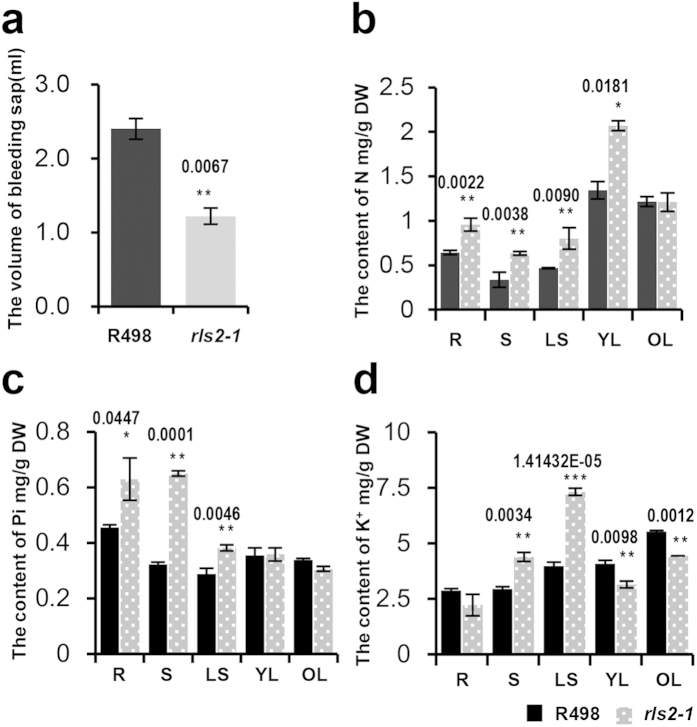
The mutation in the *rls2-1* gene resulted in a reduction in hydraulic conductance and affected primary nutrient assimilation. (**a**) The volume of bleeding sap collected from wild-type and *rls2-1*. Error bars indicate SD (n = 5). (**b**–**d**) Alterations in the concentration of primary nutrients in different tissues of wild-type and *rls2-1* mutant plants, N(**b**), Pi(**c**), K^+^ (**d**). Error bars indicate SD (n = 5). Asterisks indicate significant differences between wild-type and *rls2-1* mutant plants as determined by student’s *t*-test analysis: ***p ≤ 0.001, **p ≤ 0.01, *p ≤ 0.05.

Mutant plants showed ferruginous necrotic spots in fresh leaves, which gradually became more severe, thus leading to dried and dead leaves (Fig. 1a,b and S1). A similar symptom could be caused by inefficient water transport, excessive transpiration, and deficient mineral nutrient assimilation, particularly K^+^ deficiency. To address whether hydraulic transport or transpiration rate was affected in *rls2-1*, we performed a bleeding sap collection experiment and calculated the rate of excised-leaf water loss. Incisions were made by hand by cutting around the basilar part of a secondary internode. Plastic bags filled with absorbent cotton were affixed at the top of the culm, with the wound surface embedded in cotton. By measuring the weight at regular increments, we found that the amount of bleeding sap that was collected from the *rls2-1* mutant plants was much less than that collected from the wild-type (Fig. 2a), indicating that the *rls2* mutation affected the hydraulic transportation efficiency of stems. To ascertain whether the *rls2-1* mutant affected water transpiration, we excised fresh leaves from *rls2-1* and wild type plants at the pulvinus and weight losses were determined at different time points. No clear differences were detected, thereby indicating that *rls2-1* mutants did not develop changes in transpiration rate (Fig. S2).

Analysis of the primary nutrients in *rls2-1* and wild type plants detected changes in the levels of N, Pi, and K^+^ in *rls2-1* mutant plants in an organ-specific way. N levels were distinctively higher in the roots, stems, leaf sheaths, and young leaves of *rls2-1* mutant plants compared to those in wild-type plants, but were similar to that in old leaf tissues (Fig. 2b). A similar concentration pattern for Pi accumulation was observed, except that there were no dissimilarities in young leaf tissues (Fig. 2c). Despite the elevated levels in the roots, stems, and leaf sheath tissues (Fig. 2d), K^+^ levels dramatically decreased in leaf tissues, regardless of age or maturity (Fig. 2d). These results suggested that the *rls2* mutation affected the assimilation of primary nutrients that involves a more complex mechanism.

### Ultrastructural analyses of *rls2-1* mutants

To characterize the anatomical defects of *rls2-1* mutants, we performed semi-thin section analysis of its 2^nd^ internode of the *rls2-1*mutant. We found that the vascular bundles were smaller in *rls2-1* compared to that in the wild-type (Fig. S3a,b). The *rls2-1* mutants and wild-type plants were then compared in terms of cell width and length, which in turn contributes to the thickness and length of each internode, respectively. The width and length of the cells in the 2^nd^ internode of *rls2-1* mutants were only 59% and 82% of that in the wild-type, respectively (Fig. S3c,d). These results indicated that reduced width and length of cells at the 2^nd^ internode were related to thinner and shorter internodes in the *rls2-1* mutants.

**Figure 3:**
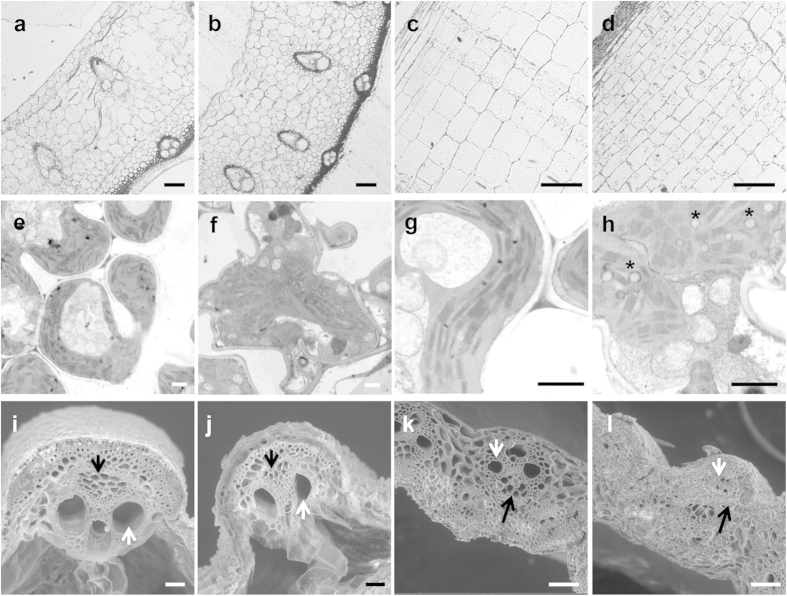
Anatomical analysis of *rls2-1* mutants. (**a**–**d**) Semi-thin section analysis of the 2^nd^ internodes of wild-type and *rls2-1* mutant plants in cross (**a**,**b**) and longitudinal sections (**c**,**d**). (**e**–**h**) TEM comparison of chloroplast cells between the wild-type (**e**,**g**) and *rls2-1* mutant plants (**f**,**h**). Large membrane-bound compartments were observed in mutant chloroplast cells as indicated by the asterisk. Bar = 3μm. (**i**–**l**) SEM observation of wild-type (**I**,**k**) and *rls2-1* (**j**,**l**) vesicular bundle in leaf veins (**i**,**j**) and stems (**k**,**l**). Xylem and phloem are indicated by white and black arrows, respectively. Bar = 100 μm in (**a**–**d**,**i**,**j**) and 3 μm in (**e**–**h**), and 200 μm in (**k**,**l**).

To assess ultrastructural alterations in *rls2-1*, we compared the chloroplast cells in the leaves, as well as vessel elements in the roots between the *rls2-1* mutants and the wild-type plants by using transmission electron microscopy. Figure 3e–h shows no clear organelle structural alterations, except for abundant vesicle body accumulation (indicated by white arrowheads) in the *rls2-1* mutants. We then observed the transection of leaves and nodes via scanning electron microscopy, which indicated that the vascular bundles of the leaves of *rls2-1* mutants were smaller than those of the wild-type, with the volume of the xylem cavity significantly reduced (Fig. 3i,j). In mature nodes, the situation was more severe, and the bundles were highly compact (Fig. 3k,l). These results demonstrated that the *rls2* mutation led to narrower vascular bundle, which possibly compromised its function in water and nutrient transport.

### Map-based cloning of *RLS2*

**Figure 4:**
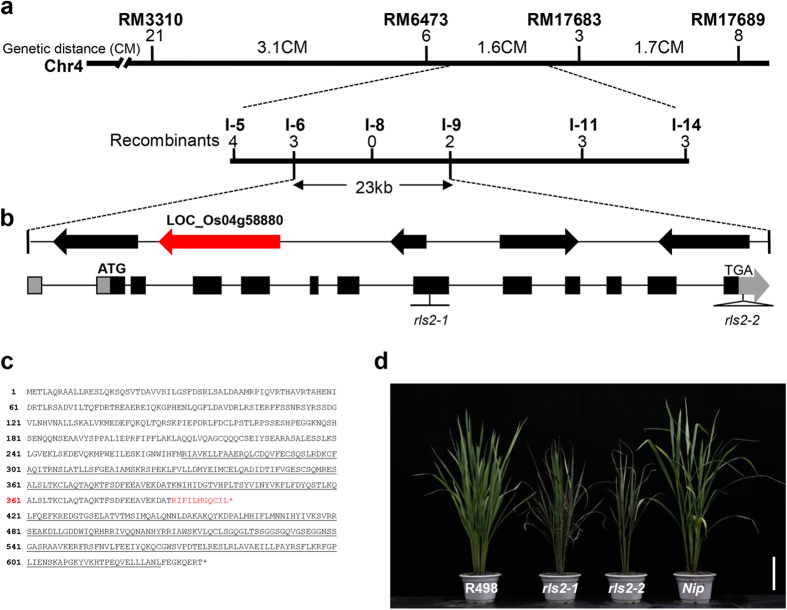
Map-based cloning of the *RLS2* gene. (**a**) Preliminary and fine mapping of the *RLS2* gene. The *RLS2* locus was preliminarily mapped to rice chromosome 4 (Chr 4) between markers RM3310 and RM17689. Then, the gene was further localized to a 23-kb genomic region between markers I-6 and I-9. cM, centiMorgan. (**b**) *RLS2* gene structure (upper panel). Gray shading, untranslated region; black shading, ORF region; Right angle, the site of single-base deletion mutation in *rls2-1*; triangle, the T-DNA insertion site of *rls2-2*. (**c**) Single-base deletion causes termination of translation in the *rls2-1* mutant. (**d**) Allelic mutant *rls2-2* exhibited similar but partially alleviated phenotype. Bar = 25cm.

To investigate the molecular basis underlying the observed alterations in *rls2-1* mutants and to identify the causative gene responsible for this particular phenotype, we performed map-based cloning of the candidate gene. First, genetic analysis of the F2 population of the *rls2-1* mutant and the wild-type (R498) plants revealed that the mutant necrotic leaf and dwarfism phenotypes were controlled by a single recessive nuclear locus (Table S1). Then, a mapping population was generated by crossing the *rls2-1* mutant with *Nipponbare*, polymorphic japonica variety; a total of 1,912 homozygous mutant plants were isolated and used for mapping analysis. The candidate gene was roughly mapped between the polymorphic molecular markers RM6473 and RM17683 on chromosome 12 (Fig. 4a). Further fine mapping using newly designed adjacent INDEL markers (Table S2) narrowed down the location of the candidate gene to a 23-kb DNA region between markers I-6 and I-9, with three and two recombinants for the two markers, respectively (Fig. 4b). Based on annotations of the rice genome database, five putative open reading frames (ORFs) were predicted within this region (Table S3). Furthermore, a newly designed INDEL marker, I-8, was localized to one of these five ORFs, whereas none of recombinants were detected in I-8. Based on these observations, we presumed that the ORF of Os04g58880, which encodes EXO70A1, was the best candidate gene that was responsible for the phenotype observed in the *RLS2* mutants. To test this hypothesis, we amplified and sequenced the ORF of Os04g58880 and found that a single nucleotide deletion in the seventh exon at nucleotide position 1,064, which is predicted to result in a frameshift mutation in *rls2-1* (Fig. 4c). These findings suggested that *Loc_Os04g58880* might be the candidate gene locus responsible for the mutant phenotypes, and was thereby designated as *RLS2*.

To further confirm that the mutation in *Loc_Os04g58880* corresponded to the *rls2* locus, we introduced a wild-type genomic DNA fragment containing *Loc_Os04g58880* into the *rls2-1* mutant to determine whether wild-type *Loc_Os04g58880* locus could complement the defective *rls2-1* phenotype. However, no positive transgenic plants were generated, possibly due to the low efficiency in genetic transformation of the *indica* genetic background of *rls2-1*. Interestingly, we obtained a loss-of-function allelic mutant line from RMD mutant libraries^44^, in which a T-DNA fragment was inserted into the 3’UTR that partially affected transcription (Fig. 4d, Fig. S4) and we named it *rls2-2*. The *rls2-2* mutants also exhibited necrotic leaves with ferruginous spots, short plant height, and lower tiller number, similar to observed in the *rls2-1* mutants (Fig. S4). Taken together, these results indicated that *Loc_Os04g58880* encodes the *RLS2* gene.

### RLS2 is widely expressed in most tissues and preferentially in differentiated vascular bundles

**Figure 5:**
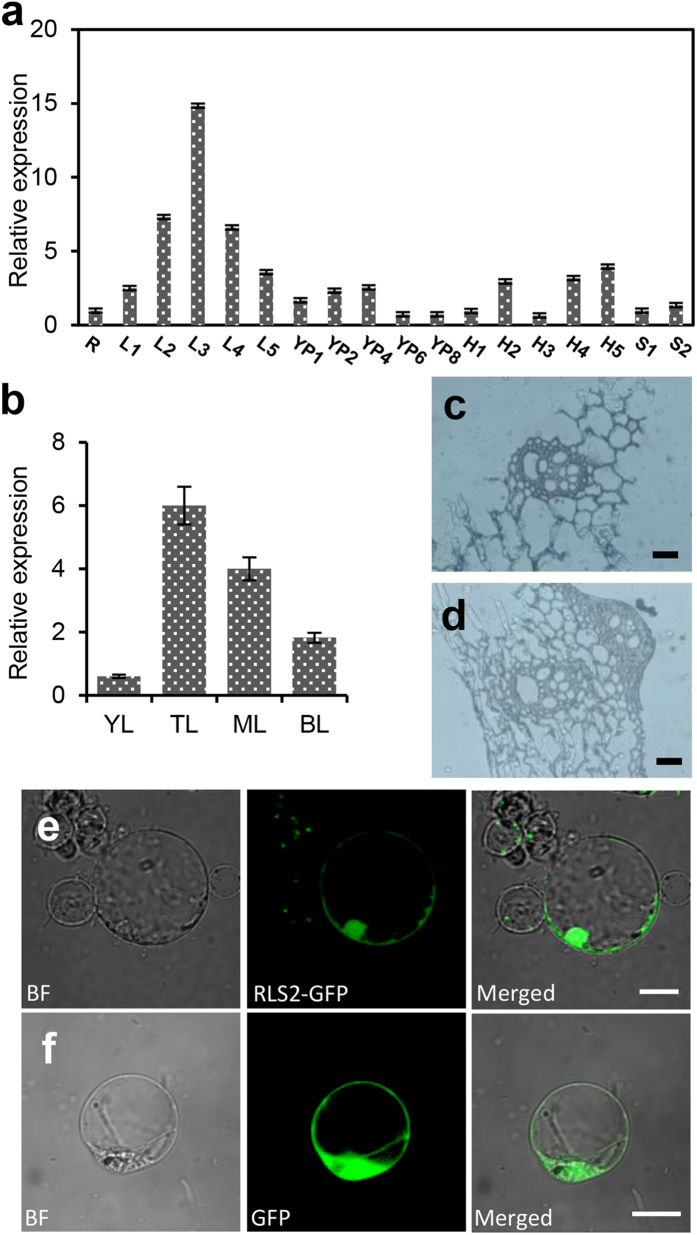
*RLS2* expression profiling and protein subcellular localization. (**a**,**b**) RT-PCR analysis of *RLS2* expression (R, seedling root; L1-L5, seedling stage, tilling stage, booting stage, heading stage, and filling stage leaves, respectively; YP1–YP8, 1 cm, 2 cm, 4 cm, 6 cm, and 8 cm young panicles, respectively; H1–H5, 1 mm, 3 mm, 5 mm, 6 mm, and 7 mm spikelets, respectively; S1–S2, tilling stage and heading stage internodes); YL, Young leaves, TL, Tip part of leaves; ML, middle part of leaves; and BL, base part of leaves. Error bars indicate SD (n = 3). (**c**,**d**) RNA *in situ* hybridization detection of *RLS2* expression in internode, antisense probe (**c**); sense probe (**d**), Scare bar = 200 μm. (**e**,**f**) Subcellular localization of OsRLS2. Transient expression of OsRLS2-GFP recombinant protein (**e**) and GFP protein (**f**) in rice protoplast cells. Scale bar = 20 μm.

To investigate the mRNA expression profile of *RLS2*, total RNA was extracted from the roots, culms, leaf blades, leaf sheaths, and inflorescences at different developmental stages. cDNAs were synthesized by reverse transcription and the expression levels were determined by real-time PCR analysis. The results showed that *RLS2* mRNA was ubiquitously expressed in most tissues, with highest level in leaves at the booting stage, followed by the spikelets; lower levels were detected in young roots, panicles, and stems (Fig. 5a). Then, we investigated whether *RLS2* was equably expressed in leaves. To answer this question, we assessed the mRNA expression of *RLS2* using different parts of the leaves. Lower expression levels were detected in young leaves, which were encapsulated by the leaf sheath and white in color (Fig. 5b), and a successive increase in expression was observed from the base to the tip of the leaves (Fig. 5b). To more precisely investigate the spatial expression pattern of *RLS2*, we performed RNA *in situ* hybridization using stem sections from wild-type plants. *RLS2* expression was detected predominantly in the stem vascular bundles (Fig. 5c,d). Together, these results indicated that *RLS2* was constitutively expressed in all tissues and was spatially restricted in vascular bundles, which in turn suggested that it might be involved in vascular morphogenesis.

To determine the subcellular location of RLS2, we prepared green fluorescent protein (GFP)-tagged construct of RLS2 and transiently expressed in rice protoplasts under the control of the 35S promoter. Punctate fluorescent signals located both on the PM and within the nucleus were observed by confocal microscopy at 16 h post-transformation (Fig. 5e). The protoplasts transformed with the empty GFP vector as control showed green fluorescent signals that were randomly distributed across the entire cell (Fig. 5f). These results suggested that the fusion protein was localized in both PM and nuclear regions.

### *rls2-1* mutants show irregular TE development and SCW deposition

**Figure 6:**
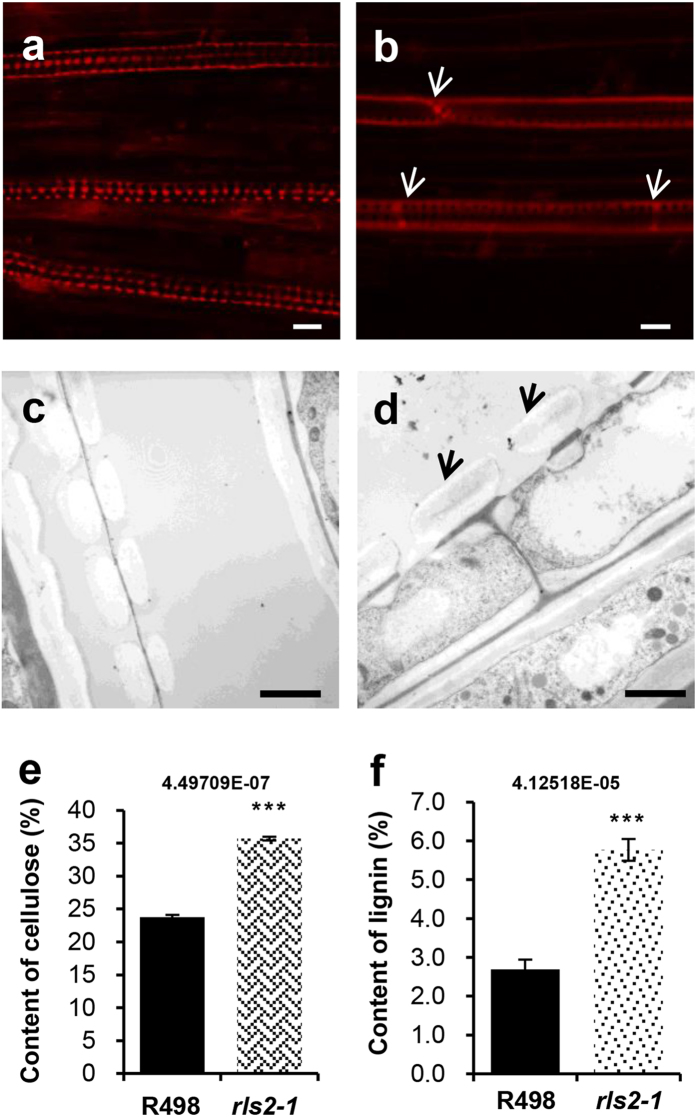
Defective TE formation in *rls2-1* mutant plants. (**a**,**b**) Mature metaxylem from wild-type (**a**) and *rls2-1* mutant (**b**) roots were stained with propidium iodide and observed under a confocal microscope. Note that pits were irregular and the perforation between two adjacent TEs in *rls2-1* was incomplete, as indicated by white arrowheads. Bar = 10 μm. (**c**,**d**) TEM images of longitudinally sectioned mature xylem in wild-type (**c**) and *rls2-1* (**d**) roots. Note the irregular SCW deposition (black arrowheads) on the inner side of TEs in the *rls2-1* mutant plants. Bars = 2.5 μm. (**e**,**f**) The contents of cellulose (**e**) and lignin (**f**) clearly decreased in the *rls2-1* mutant plant. Error bars indicate SD (n = 5). Asterisks indicate significant differences between wild-type and *rls2-1* mutant plants as determined by student’s *t*-test analysis: ***p ≤ 0.001.

Expression profiling revealed that the expression of *RLS2* was spatially restricted to vascular bundles, and anatomic observation showed vestigial vascular bundles in the leaves and stems of *rls2-1* mutant plants. We thus presumed that *RLS2* was responsible for TE formation in vascular bundles. To verify this hypothesis, we further examined the development of TEs in *rls2-1* mutants using propidium iodide-stained root samples. Confocal microscopy revealed irregular pit patterns in *rls2-1* plants compared to that of wild-type plants, which presented vessel cells with well-organized pits (Fig. 6a,b). The TEs of wild-type plants were indented or deeply lacerated, whereas that of the *rls2-1* mutants appeared smooth. The perforations between two adjacent TEs in the *rls2-1* mutants were narrower than those in the wild-type (Fig. 6a,b, indicated by white arrowheads), with a structural barrier existing at the site of the perforations, which could possibly hamper the transport of materials across the plant.

Subsequently, we examined ultrathin sections of the root elongation zone of the mutant and wild-type plants by transmission electron microscopy (TEM), which showed that TE differentiation and secondary cell wall (SCW) deposition were active. Instead of the regularly patterned SCW thickening in the wild-type plants (Fig. 6c), larger-sized SCW thickening within mature TEs were observed in the *rls2-1* mutants (Fig. 6d, indicated by black arrowheads). Interestingly, SCW thickening occurred on both sides of the TE PM and formed ribosome-like structures in the wild-type, whereas SCW thickening was only deposited on the inner side of the TE PM (Fig. 6d, indicated by a black arrowhead), which was consistent with the discovery that TEs exhibited a smooth surface in *rls2-1* mutants. The above results demonstrated that *RLS2* is essential for TE differentiation and SCW deposition.

Next, to examine whether the mutation of *rls2* generally caused comprehensive irregular SCW deposition, we evaluated the cellulose and lignin content of mature 2^nd^ internodes from the wild-type and *rls2-1* mutant plants. The cellulose and lignin content of *rls2-1* mutant internodes were predominantly higher than that observed in the wild-type (Fig. 6e,f).

### The genes involved in primary nutrients uptake and transport are regulated in a tissue-specific manner in *rls2-1* mutants

**Figure 7:**
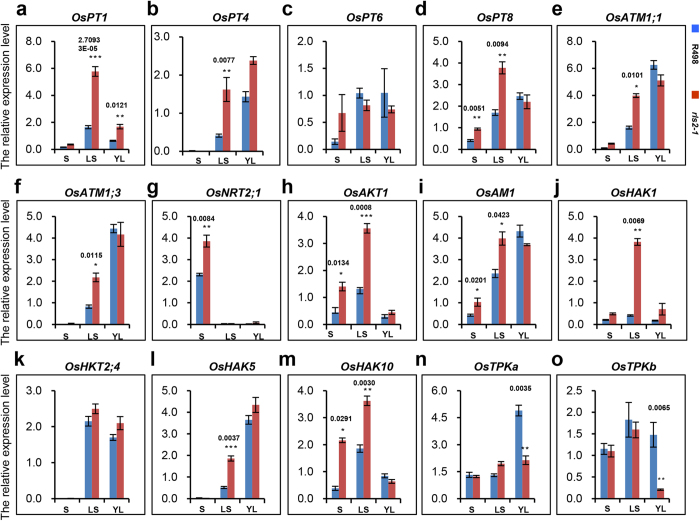
Gene expression analysis of genes involved in primary nutrient uptake, transport in wild-type and *rls2-1* mutant plants. Expression analysis of *OsPT1* (**a**), *OsPT4* (**b**), *OsPT6* (**c**), and *OsPT8* (**d**); *OsATM.1* (**e**), *OsATM.3* (**f**), and *OsNTR2.1* (**g**); *OsAKT1* (**h**), *OsAM1* (**i**), and *OsHAK1* (**j**); *OsHAK4* (**k**), *OsHAK5* (**l**), *OsHAK10* (**m**), *OsTPKa* (**n**), and *OsTPKb* (**o**) at the stems, leaf sheaths, and leaf tissues in the wild-type and *rls2-1* mutant plants by RT-qPCR. *OsActin1* was used as reference for normalization. Error bars show the SD (n = 3). S, stem tissue; LS, leaf sheath tissue; L, leaf tissue. Significant differences between wild-type and *rls2-1* mutant plants were determined by using the student’s *t*-test: ***p ≤ 0.001, **p ≤ 0.01, *p ≤ 0.05.

The mechanism of absorption, transportation, and assimilation of three primary nutrients have been well studied in both *Arabidopsis* and rice^1^,^2^,^3^,^4^,^5^,^6^,^7^, which in turn has allowed us to verify whether tissue-specific regulation of primary nutrient accumulation was associated with the altered expression of Pi, N, and K^+^ transporters or channels in the *rls2-1* mutants. The expression levels of four members of rice high-affinity phosphate transporters (OsPT1, OsPT4, OsPT6, and OsPT8)^45^, one nitrate transporter (OsNRT2.1), two ammonia transporters (OsAMT1.1 and OsAMT1.3)^11^,^46^,^47^, and eight K^+^ transporters or channels (OsAK1, OsAM1, OsHAK1, OsHAK4, OsHAK5, OsHAK10, OsTPKa, and OsTPKb)^4^,^5^,^6^,^7^ were examined by qRT-PCR analysis in the wild-type and *rls2-1* mutant plants. Consistent with the observation that the Pi and N contents were distinctively higher in stems, leaf sheaths, and leaf tissues of the *rls2-1* mutants (Fig. 2b,c), the genes responsible for the uptake and transport of Pi and N were predominantly upregulated in aerial tissues of the *rls2-1* mutants (Fig. 7a–g).

K^+^ levels simultaneously increased in the stem and leaf sheath tissues of the *rls2-1* mutant plants (Fig. 2d). Furthermore, the corresponding genes involved in K^+^ uptake and long-distance transport that encoded PM-specific proteins were upregulated in the stem and leaf sheath tissues of the *rls2-1* mutants (Fig. 7l–q). K^+^ deficiency was detected in the young leaf tissues of the *rls2-1* mutant plants (Fig. 2d), and qRT-PCR analysis revealed that the transcriptional level of tonoplast-located K^+^ channel (TPKs) was significantly reduced in the leaf tissues of the *rls2-1* mutants (Fig. 7h–m). On the other hand, no detectable discrepancies in transcript accumulation or downregulation of PM-located K^+^ transporters or channels were observed (Fig. 7n,o). These results demonstrated that the expression of K^+^ transporters or channels was regulated by *RLS2* in a highly specific manner.

### Expression analysis of EXO70A1 orthologous genes

**Figure 8:**
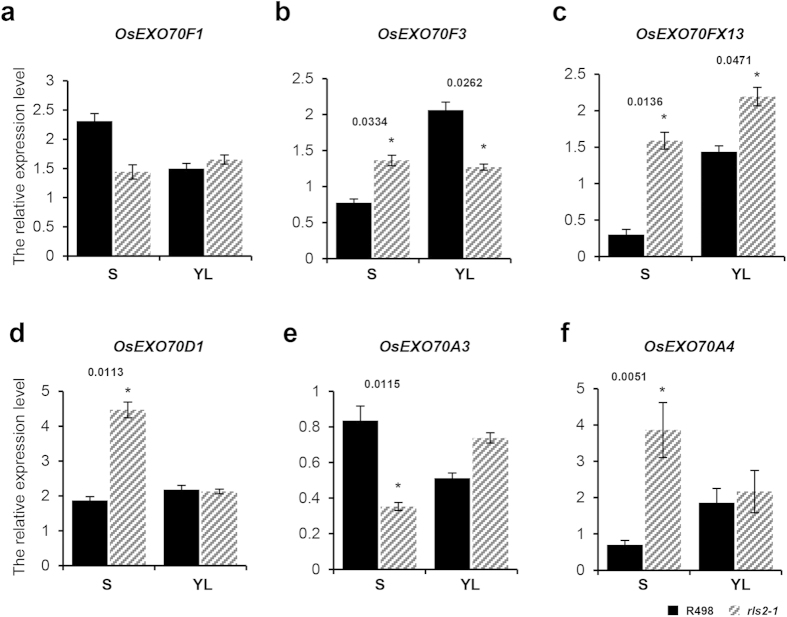
The mutation in the *rls2* genes causes transcriptional fluctuations in orthologous genes *in vivo*. (**a**–**f**) Gene expression analysis for *OsEXO70* genes in wild-type and *rls2-1* mutant plants. Expression analysis of *OsEXO70F1* (**a**), *OsEXO70F3* (**b**), *OsEXO70FX13* (**c**), *OsEXO70D1* (**d**), *OsEXO70A3* (**e**), *OsEXO70A4* (**f**) in the stems and leaves of wild-type (R498) and *rls2-1* mutant plants by RT-qPCR. *OsActin1* was used as reference for normalization. Error bars indicate SD (n = 3). S, stem tissue; L, leaf tissue. Significant differences between wild-type and *rls2-1* mutant plants were determined by using the student’s *t*-test and indicated by asterisks: *p ≤ 0.05.

The *Arabidopsis* and rice genomes encode 23 and 47 *EXO70* genes, respectively, which are then further grouped into nine clusters^28^,^32^. *EXO70A1*, *EXO70D2*, and *EXO70H7* appear to be constitutively expressed in *Arabidopsis*. Because of the absence of data on the expression and functional analyses of *EXO70* genes in rice, we resorted to using the Rice Expression Profile Database (RiceXPro) (http://ricexpro.dna.affrc.go.jp), which has revealed that the expression of four *OsEXO70* genes, namely, *OsEXO70F1*, *OsEXO70FX13* (monocot-specific Exo70s), *OsEXO70F3.* and *OsEXO70D1*, were widely interwoven with the *RLS2* protein. Three additional isoforms were also detected in the *EXO70A* cluster, which included *OsEXO70A2* (*Os04g58870*), *OsEXO70A3* (*Os11g05880*), and *OsEXO70A4* (*Os12g06270*). To investigate whether the mutation in the *rls2* gene results in changes in the transcriptional patterns of these orthologous genes *in vivo*, qRT–PCR was performed using total RNA isolated from the culms and leaves of *rls2-1* and wild-type plants. The expression levels of the *OsEXO70F3*, *OsEXO70FX13*, *OsEXO70D1*, and *OsEXO70A4* genes were markedly higher in the *rls2-1* stems (Fig. 8b–d,f). Enhanced expression levels of *OsEXO70FX13* and *OsEXO70A3* genes were also observed in the *rls2-1* leaves (Fig. 8c,e), whereas *OsEXO70F1* and *OsEXO70A3* significantly decreased in *rls2-1* stems (Fig. 8a,e). Taken together, these results revealed that the mutation in the *rls2* gene affected the expression of multiple orthologous *OsEXO70* genes *in vivo*.

## Discussion

Previous phylogenetic analysis of EXO70 protein sequences has indicated that angiosperm EXO70s could be grouped into three subfamilies and nine clusters^32^, of which the EXO70.1 proteins (including the angiosperm EXO70A cluster) are considered as the best candidates for genuine exocyst subunits. Among the mutants in this gene family, only T-DNA insertions in the *EXO70A1* gene result in a discernible phenotype in *Arabidopsis*^28^. The *exo70a1-1* mutants exhibit indeterminate highly branched inflorescence and delayed senescence, and *exo70a1-1* seedlings exhibit a higher percentage of branched root hairs when grown in liquid culture medium^28^. Even though a higher number of *EXO70* genes are encoded in rice, functional loss of *RLS2* could not fully be compensated by other paralogs. Similarly, we observed pleiotropic defects, including dwarfism, lower tiller number, and abnormal tracheary element differentiation (Figs 1a and 4a–d). Unlike the *exo70a1* mutant in *Arabidopsis*, the *rls2-1* mutant showed early senescence of leaves and normal root morphology (Fig. 1a and S1). These results indicated that compared to *Arabidopsis EXO70A1*, *RLS2* possesses a highly conserved and distinct function in rice.

Previous studies have revealed that cortical microtubules and the actin cytoskeleton play essential roles in the trafficking of vesicles that carry various kinds of protein complexes and materials to the PM, where the secondary wall was deposited^48^. It has been proposed that the *EXO70A1* gene functions in vesicle trafficking during vessel differentiation^37^. The present study determined that *rls2-1* mutants possessed vessel cells with thicker ectopic SCW and significantly higher cellulose and lignin levels (Fig. 4c,d). In addition, we have determined that some *EXO70* orthologous genes were differentially expressed in *rls2-1* mutants. The expression of *OsEXO70F3, OsEXO70FX13, OsEXO70D1,* and *OsEXO70A4* genes markedly increased in *rls2-1* stems (Fig. 8a–f). Based on these findings, we hereby propose two hypotheses that could potentially explain the observed thickening of the SCW in *rls2-1* mutants. (1) Vesicle trafficking not only carries the CesA synthesis complex and hemicellulose that are utilized in cell wall formation, but also transports cellulase and hemicellulase that are essential for cell wall remodeling during the cell extension stage^49^,^50^. These processes are facilitated by *EXO70A1*, which is supported by the fact that cell size in transverse and longitudinal directions significantly decreased in the mutants (Fig. 3a–d and S3). (2) The EXO70 subunit facilitates plant cell-specific exocyst formation and potentially functional divergence^28^,^29^. The increased expression of EXO70 orthologous genes in the stems of *rls2-1* mutants possibly induces the assembly of a distinct exocyst complex, which in turn disrupts normal SCW deposition, thereby causing disorders in vesicle trafficking.

Most importantly, we found that the mutation in the *rls2* gene affects the assimilation of primary nutrients. N, Pi, and K^+^ levels were regulated in a tissue-specific manner in the *rls2-1* mutants (Fig. 2b–d). Therefore, the excessive accumulations of N, Pi, and K^+^ in the roots and leaf sheath tissues resulted in *rls2-1* lethality. Young leaves showed severe K^+^ deficiency, which is the main cause of ferruginous necrotic spots in *rls2* mutants. We were not convinced that most of the defects observed in the *exo70a1-1* mutant could be simply explained by imperfect TEs in *Arabidopsis*^37^.

In the Pi signaling pathway, ubiquitination mediated by PHO2 (E2 and LTN1 in rice) and NLA (E3) play key roles in the removal of proteins from the PM via the endocytic pathway^51^. The ubiquitination of PHT1s in post-ER compartments is mediated by PHO2, whereas NLA-dependent ubiquitination is required for clathrin-dependent endocytosis of the PM-localized PHT1s^8^,^9^,^52^. The fact that neither PHO2 nor NLA have been predicted to have any transmembrane domain or post-translational lipid modification raises questions about the mechanism underlying its recruitment to the PM. We found that the high-affinity transporters of Pi and N are uniformly upregulated in *rls2-1* mutants (Fig. 7a–g), which in turn may lead to the accumulation of Pi and N in various plant tissues. These results suggest that exocysts are involved in membrane-associated ubiquitin machinery, by which PHO2 and NLA are recruited to the PM. Pi and N high-affinity transporters are usually located in the PM, whereas K^+^ transporters or channels are found not only in the PM, but also in other membrane systems^5^,^7^,^15^. In particular, the tonoplast-located K^+^ channels are necessary for maintaining cellular electroneutrality and osmotic equilibrium^5^. However, little is known about the mechanistic details of how integral membrane proteins arrive at its specific destinations. Here, TM-located K^+^ transporters (*OsAK1*, *OsAM1*, *OsHAK1*, *OsHAK4*, *OsHAK5*, and *OsHAK10*) and tonoplast-located K^+^ channels (*TPKa* and *TPKb*) were discriminatively regulated in *rls2-1* mutants (Fig. 7h–o). The former were uniformly upregulated in the stems and leaf sheath tissues of the *rls2-1* mutant plants, whereas these were minimally affected in leaf tissues (Fig. 7h–m). On the other hand, the latter were distinctively repressed in the leaf tissues of *rls2-1* mutants and normally expressed in the other tissues (Fig. 7n,o). In summary, our results have revealed that *RLS2*-mediated vesicle trafficking could be involved in the distribution of tonoplast-located K^+^ to specific membrane destinations. However, further investigation is needed to be performed to understand the functional complexity of *EXO70A1* in rice and *Arabidopsis*.

## Materials and methods

### Plant materials and growth conditions

Rice (*Oryza sativa*) mutant *rls2-1* was isolated from the *indica* cultivar EMS-mutated R498 population, an elite restorer line for hybrid rice. All plants were grown in paddy fields of the Rice Research Institute of Sichuan Agriculture University (China, Cheng Du) or Ling Shui (Hainan Province, China) during its natural growing season.

### Measurement of primary nutrient concentrations

Samples from different tissues of the *rls2-1* and wild-type plants were collected at the booting stage, rinsed with deionized water, and dried at 80 °C to constant weight in paper bags. The dry weights of the samples were measured as dry biomass. For the measurement of total P concentration, ~0.05-g of the dry samples was used following in the method described elsewhere^53^. N concentration was measured using a colorimetric method^45^. K^+^ content analysis was conducted as previously described^6^.

### Bleeding sap collection and rate of water loss in excised leaves

Bleeding sap collections were performed as described elsewhere^54^. Generally, bleeding sap collection was performed on plants grown in the paddy fields; it involved the 12-h direct collection of sap from a stem that was cut off 9–10 cm above the soil surface into the provided cotton. The bleeding sap volume was calculated from the weight increase of the cotton. For the excised-leaf water loss rate and fresh flag leaves of main tillers were excised from *rls2-1* and wild-type plants at the position of the pulvinus. Leaf area was measured by using a living leaf area meter (YMJ-B). Weight loss was recorded at distinct time points. The excised-leaf water rate was calculated based on the weight loss at each time point versus leaf area.

### Anatomical analyses

To evaluate cell anatomical features of the plants, fresh hand-cut sections (approximately 20 μm) of the 2^nd^ internode of culms and 2nd leaves were prepared and stained with toluidine blue and examined under a light microscope (Leica).

SEM was used as previously described^55^; samples at different stages were pre-fixed in a 0.1 M sodium phosphate buffer containing 2.5% glutaraldehyde (pH 6.8) overnight at 4 °C, then rinsed twice with 0.1 M phosphate buffer (pH 6.8). The samples were dehydrated using an acetone series of increasing gradations from 30% to 100%, and then exchanged three times with isoamyl acetate. The fixed samples were processed for critical-point drying using liquid CO_2_, and then gold coated. The samples were examined under a JEM-1200 EX scanning electron microscope (Hitachi).

For TEM, samples from leaves, stems, and roots of *rls2-1* plants and wild-type plants were collected. The tissues were prefixed in 3% glutaraldehyde, and then fixed in a 0.1 M sodium phosphate buffer containing 2% osmium tetroxide for at least overnight at 4 °C. The tissues were then dehydrated in an acetone series, infiltrated in Epox 812 for 4 h, and embedded. Ultrathin sections were cut with a diamond knife and stained with uranyl acetate and lead citrate. Sections were examined under a transmission electron microscope (HITACHI, H-600IV, Japan).

### Map-based cloning of *RLS2*

The *rls2-1* mutants were crossed with Nipponbare, a polymorphic japonica variety that has been widely used for map-based cloning. In the F2 progeny, plants showing necrotic spotted leaves and dwarfism were selected for gene linkage analysis. Preliminary mapping was performed with molecular markers distributed across the 12 rice chromosomes. Fine-mapping sequence-tagged site primers were designed according to the different DNA sequences of *indica* and *japonica* (http://www.ncbi.nlm.nih.gov). Primers used in fine mapping are listed in Supplemental Table S2.

### RNA extraction and qRT-PCR

RNA extraction and qRT-PCR were performed as previously described^56^. mRNA was extracted from the collected tissue samples using TRIzol (Invitrogen, Carlsbad, CA, USA). About 1000 ng of total RNA was treated with DNaseI and used in first-strand cDNA synthesis with oligo(dT)18 as primer. SuperScript II (Invitrogen, USA) was used as the reverse transcription enzyme. The qRT-PCR was run on CFX96™ Real-Time System (Bio-Rad, USA) with gene-specific primers. The reaction was performed at 95 °C for 1 min, 40 cycles of 95 °C for 10 s, and 60 °C for 30 s. The sequences of the primers used are listed in Table S2. Rice *ACTIN1* was used as internal control in all analyses. The ΔΔCq method of the CFX Manager™ software version 3.0 (Bio-Rad, USA) was used to normalize gene expression. Three replicates were performed for each gene.

### *In situ* hybridization

*In situ* hybridizations were performed as described elsewhere^55^, with minor modifications. To generate *RLS2*-specific sense and antisense probes, a 455-bp fragment at the 3’-UTR of the full-length cDNA was amplified from wild-type cDNA using *RLS2*-specific primers, both at the 3’-end and 5’-end, of which a T7 promoter binding sequence (TAACTAATACGACTCACTATAGGG) was added and then transcribed with a T7 RNA polymerase. The primers are listed in Table S2.

### Subcellular localization

Rice protoplasts were isolated from Nipponbare seedlings and were transformed with the recombinant plasmids pC2300-*RLS2* and pC2300 alone as described elsewhere^57^. Fluorescence was examined under confocal microscopy (Nikon A1 i90, LSCM, Japan) at 16 h post-transformation.

### Measurement of cellulose and lignin levels

The 2^nd^ internode tissues of *rls2-1* and wild-type plants were collected at the filling stage, rinsed with deionized water, heated at 105 °C for 0.5 hour, and then dried at 80 °C to constant weight in paper bags. The dried samples were fully ground and passed through an 80-mesh size seize. The measurement of cellulose and lignin were performed using Fibertec^TM^ M6 1020 (FOSS).

### Plasmid construction

For the construction of the *pACTIN::RLS2*-*GFP* plasmid, cDNA (without the stop codon) was PCR amplified using primers *RLS2*-CDSF and *RLS2*-CDSR and inserted to into plasmid PC2300-Actin (35S promoter was replaced by an *actin* promoter) at the *Kpn*I and *Sal*I sites. The plasmid was used to transform a Kitaake callus with *Agrobacterium* (*EHA105*). The genetic transformation of rice was performed as described by Cheng^58^. After selection for G418 resistance, the regenerated plants were confirmed by PCR-based genotyping using a primer pair specific for the *Npt* II gene (Table S2).

### Statistical analysis

The data were analyzed using the Excel software (Microsoft) for average values, SD, and student’s t-test analyses.

## Additional Information

**How to cite this article**: Tu, B. *et al.* Disruption of *OsEXO70A1* Causes Irregular Vascular Bundles and Perturbs Mineral Nutrient Assimilation in Rice. *Sci. Rep.* **5**, 18609; doi: 10.1038/srep18609 (2015).

## Supplementary Material

Supplementary Information
