# Supplementary material for: Disruption of OsEXO70A1 Causes Irregular Vascular Bundles and Perturbs Mineral Nutrient Assimilation in Rice
Source: Sci Rep. 2015 Dec 22;5:18609. doi: 10.1038/srep18609 (PMC4686888; doi:10.1038/srep18609)

Disruption of OsEXO70A1 Causes Irregular Vascular Bundles and Perturbs Mineral Nutrients Assimilation in Rice

Bin Tu[1,2#], Li Hu[1#], Weilan Chen[1,2#], Tao Li[1], Binhua Hu[1], Ling Zheng[3], Zheng Lv[1], Shuju You[1,4], Yuping Wang[1,2], Bingtian Ma [1], Xuewei Chen[1], Peng Qin[1,2 *] and Shigui Li[1,2]*

Legends for Supplemental Figures

Fig S1. (a-d) Performance of whole plants in different developmental stage. Seedling stage (a), Tiller stage (b), Booting stage (c), Heading stage (d). Bar=25cm. (e) The comparison of root system between wild type and *rls2-1*. Bar=2cm. (f) Quantification of the wild-type and *rls2-1* internode length, 1nd – 5nd, the 1st to 5th internodes. Error bars indicate SD (n= 15).

Figure S2. The rate of water loss of excised-leaves between *rls2-1* mutant plants and wild type plants. Error bars indicate SD (n= 5).

Figure S3. The comparison of 2nd internode cell size between wild type and *rls2-1*. (a-b) The size comparison of vascular bundles. The length of major axis and minor axis were compared in (a) and (b). Error bars indicate SD(n=10). (c)The comparison of cell length. Error bars indicate SD (n= 63). (d) The comparison of cell width. Error bars indicate SD (n= 66). p values determined by student t-test using R498 as control, *** $p \leqslant 0.001$.

Figure S4. The characterization of allelic mutant *rls2-2*. (a) *rls2-2* exhibits necrotic leaves. Bar=2cm. (b) RT-PCR to examine the *OSEXO70A1* transcript in wild type and *rls2-2*. Os*ACTIN1* was used as a loading control. p values determined by student t-test using R498 as control, *** $p \leqslant 0.001$, ** $p \leqslant 0.01$.

Table S1. Genetic analysis of *rls2-1*

Cross	Population of F2	Normal plants	Mutant plants	χ^2 (3:1)
**R498 X *rls2-1***	178	133	45	$0.172 < \chi^2_{0.05,1}$

Note: $\chi^2_{0.05,1}=3.84$

Table S2. Sequences of DNA oligonucleotides used in this study

I5 F	CCTTCTCCTTCTCGCACTC
I5 R	CCAACAACTGAACCTACCCT
I6 F	ATCCTCGTTTGGTCTGTC
I6 R	TAGGACCTAGAATACTCTTTGG
I8 F	TGGCCTCCAATCGTCTGC
I8 R	TGGGCTGGATGCGTGAAT
I9 F	CGGATGCGTCGTCTACTA
I9 R	TACACCCGCAATTCCATC
I11 F	ACTACACGAGTGAGCTGGAC
I11 R	TAGCAGCTAAGAGCAAGGAT
I14 F	GTGTTCTTTGCCCAGGGTT
I14 R	ACCGGAACAGTGATCGTG
rls2-2 LP	TTACCGAAGTCACCCATGTG
rls2-2 RP	CTTTGGTGCATACCACATGC
RMD-R	GCCTCAAGAAGCTCAAGTGC
RMD-L	ATAGCTGGGCAATGGAATCCG
Insitu T7&F	TAACTAATACGACTCACTATAGGGCTGTCCGCAGATCAGAAGCCA
Insitu F	CTGTCCGCAGATCAGAAGCCA
Insitu T7&R	TAACTAATACGACTCACTATAGGGTCAAGTACGCTCTTGTTTTCC
Insitu R	TCAAGTACGCTCTTGTTTTCC
RLS2 CDS-F	CGGGGTACCATGGAGACCCTTGCGCAGCGC
RLS2 CDS-R	CGCGTCGACAGTACGCTCTTGTTTTCCTTC
RLS2-RTF	AAAAGCAGTGTGGCTGGTCT
RLS2-RTR	TATTTTCCGGGTGCCTTGCT
OsPT1RT-F	AGCGTTCGGGTTCCTGTA
OsPT1-RTR	CGTTCTTGATGCCGATCC
OsPT2-RTF	CACAAACTTCCTCGGTATGCT
OsPT2-RTR	GAAACCCCACAAATCCACAAC
OsPT4-RTF	TTCTGCTAGTGTACCAAACAAAATTACA
OsPT4-RTR	CTAAGTGGCATTTATAATATCAACAGTAACC

OsPT6RTF	GCCCCTGCAAACTGTACTG
OsPT6-RTR	AGCCAGGCCAGTTATATATCAAC
OsPT8-RTF	CCTACTTGTGTTTGTCTATGTG
OsPT8-RTR	GTGCCAAATTGCTGGTCTG
AM1	GGCTGTGGTGGTCCTGTTGA
AM1	ACAGACGACCTCCAGCGATTAT
OsAKT1-RTF	TACGACCGCCGATACAGAA
OsAKT1-RTR	CCAAATAAGCCACAAAGAAGG
OsHAK1-RTF	CAAGAGGATCGCGGTGAACTACA
OsHAK1-RTR	CTTGAGCAGCTGATCGTTTGGA
OsHAK4-RTF	CGTTCCCATCCGTCAGTAAA
OsHAK4-RTR	CAGCCTCTGGTCTGGTTCGTC
OsHAK5-RTF	CATTGTGGACTATTTTGAAAGAA
OsHAK5-RTR	GGAGAACTACAGAAAAGCCAATC
OsHAK10-RTF	CGCTCTCGGCTGCTTTCCT
OsHAK10-RTR	TAACCGCCAATCCTGACGC
OsTPKa-RTF	GAGATCTACACCGAGCGACG
OsTPKa-RTR	GAGTGGTCGACATCGAGCTT
OsTPKb-RTF	TACCTCGTCGAGAAGCAGGAG
OsTPKb-RTR	TGTAGAGCTTGTACCTCACCTTG
OsEXO70F1-RTF	TACAGGGGGAGACATCTCGG
OsEXO70F1-RTR	AGTATGAGCACACACTGCCC
OsEXO70F3-RTF	GGAGAGCTTGTCCCCACTTG
OsEXO70F3-RTR	TTTTGCCGAATCTTCCCACG
OsEXO70FX13-RTF	AGCTTGATACCGCTATGGGC
OsEXO70FX13-RTR	TCATAGAAGAGACTGCTCAACAT
OsEXO70D1-RTF	CCATCCATCCTCTCACTCGC
OsEXO70D1-RTR	GGTCGGCAAGGTCAAGATCA

Table S3. The predicted candidate genes

(Locus identifier)	(CDS length)	(protein length)	（predicted function)
LOC_Os04g58870	1572bp	524aa	Exo70 exocyst complex subunit family protein
LOC_Os04g58880	1905bp	635aa	Exo70 exocyst complex subunit family protein
LOC_Os04g58890	846bp	282aa	Conserved hypothetical protein
LOC_Os04g58900	681bp	227aa	Similar to Diadenosine 5',5'''-P1,P4-tetraphosphate hydrolase
LOC_Os04g58910	2817bp	939aa	Similar to Receptor-like protein kinase-like protein

Figure S1.

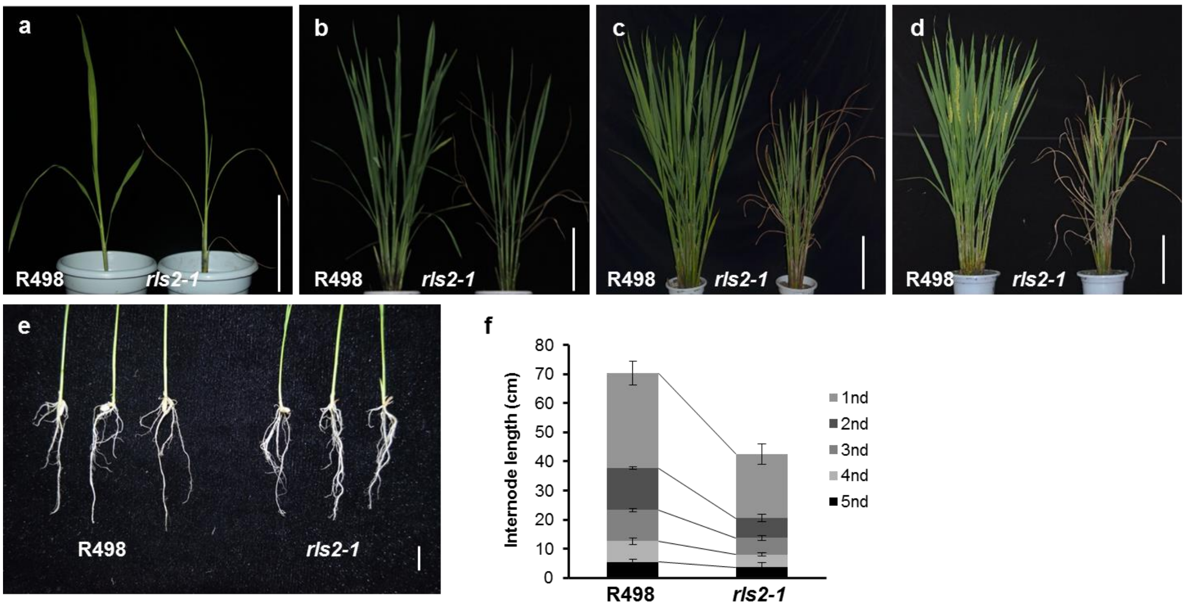

Figure S2

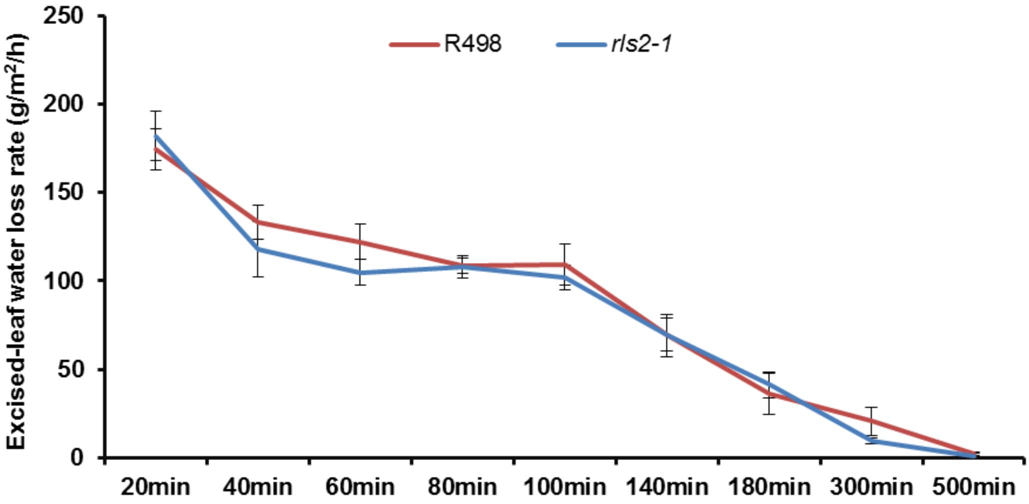

Figure S3.

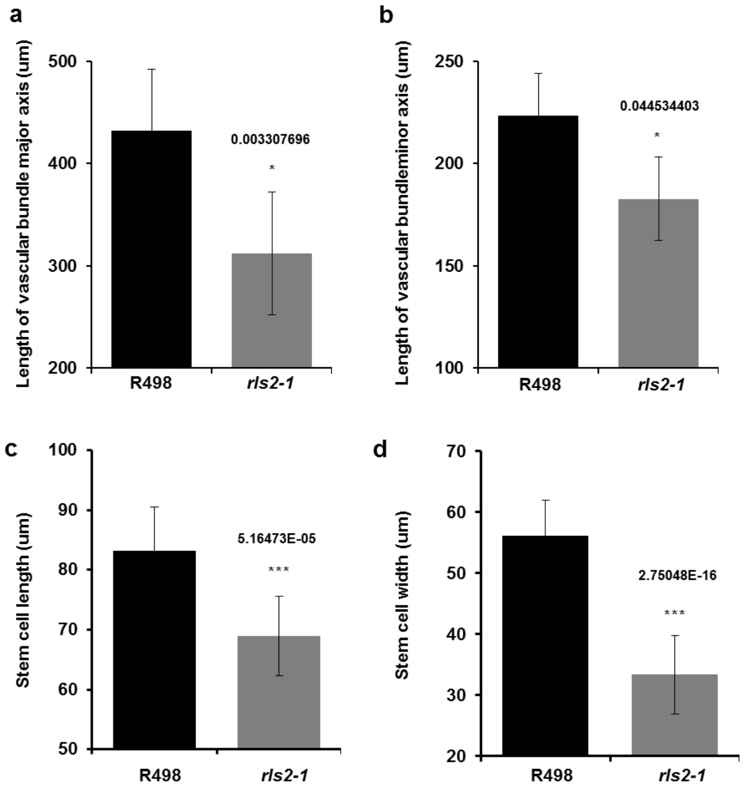

Figure S4.

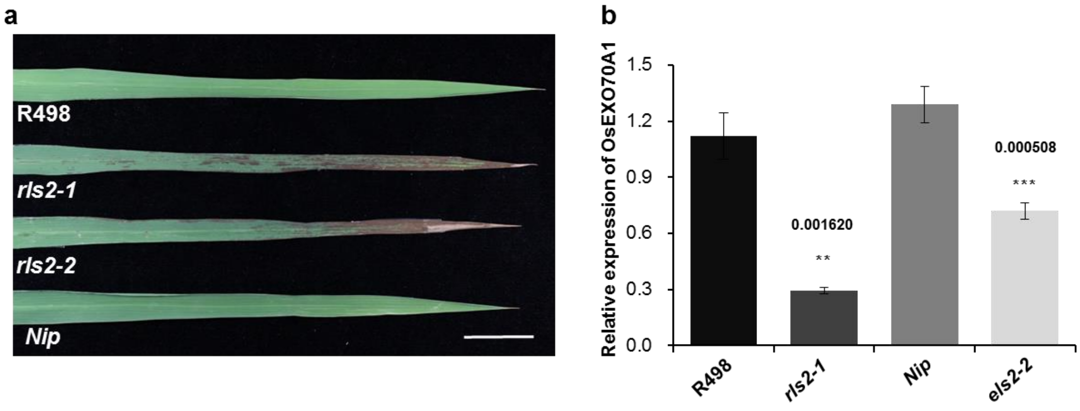

Supplement: Supplementary Information [file srep18609-s1.pdf]
